# The Influence of Elementary Silver Versus Titanium on Osteoblasts Behaviour In Vitro Using Human Osteosarcoma Cell Lines

**DOI:** 10.1155/2007/26539

**Published:** 2007-07-11

**Authors:** Jendrik Hardes, Arne Streitburger, Helmut Ahrens, Thomas Nusselt, Carsten Gebert, Winfried Winkelmann, Achim Battmann, Georg Gosheger

**Affiliations:** ^1^Department of Orthopaedics, University Clinics of Muenster, Albert-Schweitzer Street 33, 49149 Muenster, Germany; ^2^Institute of Pathology, University Clinic of Giessen and Marburg, Langhansstrasse 10, 35385 Giessen, Germany

## Abstract

*Purpose*. The antimicrobial effect of a silver-coated tumor endoprosthesis has been proven in clinical and experimental trials. However, in the literature there are no reports concerning the effect of elementary silver on osteoblast behaviour. Therefore, the prosthetic stem was not silver-coated because of concerns regarding a possible inhibition of the osseointegration. The aim of the present study was to investigate the effect of 5–25 mg of elementary silver in comparison to Ti-6Al-4V on human osteosarcoma cell lines (HOS-58, SAOS). *Methods*. Cell viability was determined by measuring the MTT proliferation rate. Cell function was studied by measuring alkaline phosphatase (AP) activity and osteocalcine production. *Results*. In the HOS-58 cells, the AP activity was statistically significant (*P* < 0.05) higher at a supplement of 5–10 mg of silver than of Ti-6 Al-4V at the same doses. For both cell lines, a supplement above 10 mg of silver resulted in a reduced AP activity in comparision to the Ti-6 Al-4V group, but a statistically significant difference (*P* < 0.05) was observed at a dose of 25 mg for the SAOS cells only. At doses of 20–25 mg in the HOS-58 cells and 10–25 mg in the SAOS cells, the reduction of the proliferation rate by silver was statistically significant (*P* < 0.05) compared to the Ti-6 Al-4V supplement. *Discussion*. In conclusion, elementary silver exhibits no cytotoxicity at low concentrations. In contrast, it seems to be superior to Ti-6 Al-4V concerning the stimulation of osteogenic maturation at these concentrations, whereas at higher doses it causes the known cytotoxic properties.

## 1. INTRODUCTION

The antimicrobial effect of silver-coated medical devices has been reported in previous studies [1–3]. Silver is in comparison to other metals low cytotoxic [4]. Toxic side effects have been described for blood concentrations of 300 ppb in form of argyrosis, leukopenia, liver, and kindney damage [1, 3]. However, systemic or localized cytotoxic side effects have been reported in doses below 300 ppb [5]. Inhibition of the proliferation of keratinocytes and fibroblasts after treatment with silver-sulfadiazin have been described [6, 7]. There are some reports describing a chronic inflammatory reaction in patients treated by a silver-coated heart valve. The authors assume a toxic reaction deriving from the silver released from the impregnated sewing cuff, which may lead to an inhibition of normal fibroblast response [8]. Nevertheless, with the use of silver one has to consider its dose-dependent cytotoxic side effects [9]. According to this, other authors have described no cytotoxicity of silver-coated medical devices [3, 10–12].

A silver coating of orthopedic medical devices in humans has not been described before. In a previous animal trial the antimicrobial efficacy and the absence of histologically proven cytotoxic side effects of a silver-coated diaphseal implant in a rabbit have been reported [9]. Nevertheless, the used prosthetic stem was not silver coated because of concerns regarding a possible inhibition of osteoblasts by silver.

To our knowledge the effect of elementary silver on bone matrix producing cells has not been investigated until now. To observe possible dose-dependent effects of silver on osteoblasts, the MTT rate of two osteosarcoma cell lines representing different stages of osteogenic maturation has been investigated. Further osteoblast function has been determined by measuring the AP activity and the osteocalcine production after addition of 5 mg to 25 mg silver powder in comparision to 5 mg to 25 mg of Ti-6Al-4V powder.

## 2. MATERIALS AND METHODS

In the current study five cell culture series and one control series have been used for the silver group and the Ti-6Al-4V group, respectively. In the silver group a silver powder supplement has been added in concentrations ranged from 5 mg to 25 mg in 5 mg steps. In the titianium group a Ti-6Al-4V powder supplement has been added in the same manner. All powder supplements have been of the same particle size of 90*μμ*m or less. The incubation period was 48 hours. All trials have been performed four times in each individual cell culture series. The reported values represent the mean value of the four trials.

### 2.1. Cell cultures

HOS 58 is an established osteosarcoma cell line, originally isoloated from an osteosarcoma of a 21-year-old man (Institute of Pathology, University of Giessen) [13]. It is characterized by a comparatively low proliferation rate. SAOS 2 (11 years, female) is in contrast to the previous one characterized by a high proliferation rate and is known to be capable of bone production [14].

Cells have been maintained in ISCOVE medium with a supplement of 10% of FCS (Gibco), 100 IU/ml of penicillin, 100 *μ*g/ml of streptomycin in a humidified atmosphere of 95% air, and 5% CO_2_ at 37°C.

In each individual cell culture, cells have been cultivated at a starting density of 20 000 cells/well in six well plates (Nunc). The cells have been placed on the bottom and have been incubated for 24 hours. The supplements have been added and build up a layer onto the cell culture.

### 2.2. Alkaline phosphatase

The AP activity has been measured with the protein assay of Lowry (p-nitrophenylphosphate assay, 37 C, 405 nm, Triton-X-100). The specific activity has been calculated as u/*μ*m protein.

### 2.3. Osteocalcin

The production of osteocalcine has been measured with the IBL-osteocalcin kit (IBL Hamburg). In this test monoclonal osteocalcine antibodies have been used. These antibodies have been detected by the use of HRP conjugated link antibodies (measurement at 405 nm).

### 2.4. Cell proliferation rate

The proliferation rate has been considered with the 3-[4,5-dimethylthiazol-2-yl]-2,5-diphenyltetrazolium bromide assay (MTT). In this test the mitochondrial activity has been measured by splitting tetrazolium salts with mitochondrial dehydrogenases in living cells only. The optical density has been read with a Microplate Reader BIO-RAD Model 550 (Bio-Rad Laboratories) at 540 nm, and with the use of a reference wavelength at 690 nm.

### 2.5. Statistical analysis

The statistical evaluation has been performed with the programs Excel and Statistical Package for Social Sciences (10.0). To determine differences in the silver-, Ti-6Al-4V- and control group, the Mann-Whitney-*U*-test has been used.

## 3. RESULTS

### 3.1. Alkaline phosphatase activity

**Figure 1:**
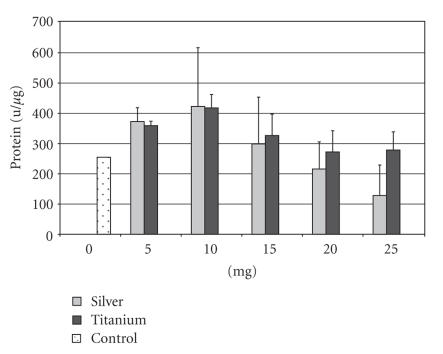
Alkaline phosphatase activity of the SAOS cell line after supplementation of 5–25 mg silver or titanium.

In the control group the AP activity was 254.7 u/*μ*m for the SAOS cell line. The supplement of 5–10 mg of silver resulted in an increase of the AP activity to 422.9 u/*μ*m. The activity at these silver doses was higher than with a Ti-6Al-4V supplement, but not statistically different. Even with a supplement of 15 mg of silver the activity was above the value of the control group with 298.8 u/*μ*m. With a supplement of 25 mg the activity decreased to 128.2 u/*μ*m (see Figure 1). The Ti-6Al-4V supplement resulted in an comparable activity increase to 418.0 u/*μ*m at 10 mg, but with only a slight decrease in concentrations above 10 mg. With a supplement of 25 mg, the activity decreased to 278.6 u/*μ*m (see Figure 1). This activity is still above the value of the control group. Statistically significant differences (*P* < 0.5) between the two groups could be observed with a supplementation of 25 mg silver/Ti-6Al-4V only.

**Figure 2:**
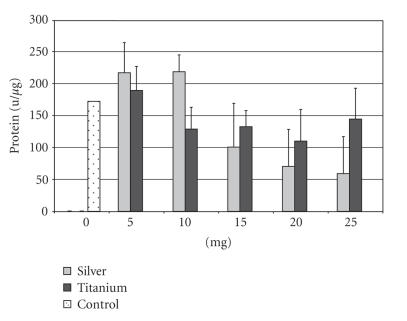
Alkaline phosphatase activity of the HOS-58 cell line after supplementation of 5–25 mg silver or titanium.

The AP activity in the control group was 172.3 u/*μ*m for the HOS-58 cell line. The supplement of 5–10 mg of silver resulted in an increase of the AP activity to 218.6 u/*μ*m. The supplement of 15 mg of silver leads to a more pronounced decrease (100.6 u/*μ*m) than in the SAOS cell line. With a supplement of 25 mg the activity decreased to 59.0 u/*μ*m (see Figure 2). The Ti-6Al-4V supplement resulted in an increased activity (189.2 u/*μ*m) at 5 mg, whereas with the supplement of 10 mg the activity (128.7 u/*μ*m) was below the value of the control group. The supplement of more than 10 mg resulted in slight activity differences only (see Figure 2). A silver supplement of 5 mg and 10 mg resulted in a significant higher AP activity than the supplement of Ti-6Al-4V at the same doses (*P* < .05).

### 3.2. Osteocalcine activity

In contrast to the AP activity there were no statictically significant differences between the silver and Ti-6Al-4V supplement. In the control series the mean osteocalcine concentration was 26.5 ng/ml for the SAOS cell line. In the series with the SAOS cell line the osteocalcine concentration increased from 26.6 ng/ml (5 mg of silver) to 27.1 ng/ml (25 mg of silver), but never reached the concentration of the control group. With the Ti-6Al-4V supplement the osteocalcine concentration was 27.1 ng/ml at 5 mg and 25.6 ng/ml at 25 mg.

In the control series the mean osteocalcine concentration was 27.3 ng/ml for the HOS-58 cell line. The osteocalcine concentration was 26.0 ng/ml at 5mg silver supplement and increased to 28.0 ng/ml at 25 mg silver supplement. With the Ti-6Al-4V supplement the concentration was 27.6 ng/ml (5 mg) and 26.3 ng/ml (25 mg), respectively.

### 3.3. Cell proliferation rate

**Figure 3:**
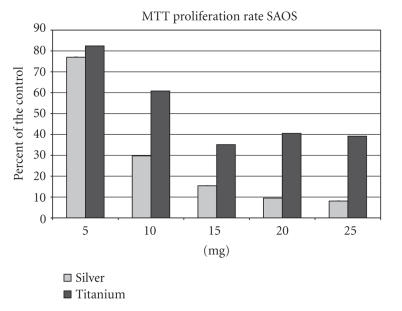
MTT rate of the SAOS cell line after supplementation of 5–25 mg silver or titanium.

In the SAOS group, the MTT proliferation rate decreased from 77.0% (5 mg of silver) to 5.4% (15 mg) and showed a slight increase in concentrations of 20 mg (9.5%) and 25 mg (8.1%) in comparision to the control group (100%) (see Figure 3). A decrease of the proliferation rate with increased Ti-6Al-4V concentrations was measured also. However, the proliferation rate was throughout higher than in the silver group with a maximum value of 82.4% (5 mg). With a supplement of 25 mg the proliferation rate was still 39.2% in comparision to the control group. As in the silver group the lowest proliferation rate was measured with a supplement of 15 mg (35.1%) and showed an increase to 40.5% (20 mg). In general, the differences between a silver and Ti-6Al-4V supplement were statistically significant at concentrations higher than 5 mg (*P* < 0.5).

**Figure 4:**
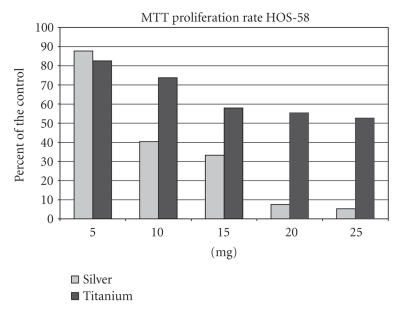
MTT rate of the HOS-58 cell line after supplementation of 5–25 mg silver or titanium.

In the silver group the proliferation rate of the HOS-58 cell line decreased continuously from 87.7% (5 mg) to 5.3% (25 mg) (see Figure 4). The proliferation rate in the Ti-6Al-4V group was obviously less reduced than in the silver group with a MTT rate of 82.5% (5 mg) to 52.6% (25 mg). Statistically differences (*P* < 0.5) in the proliferation rate between the two groups were seen at 20 mg and 25 mg supplements only.

## 4. DISCUSSION

Infection is the most common and serious complication in the reconstruction of large bone defects in tumor and revision surgery by megaendoprostheses. In literature infection rates between 8% and 13% have been reported [15–17]. Secondary amputation or hip disarticulation is in some cases the only solution [18, 19].

Silver coating of medical devices has proven antibacterial activity in vitro and in vivo [1–3, 20]. Elementary silver exhibits a dose-dependent cytotoxity [9]. According to this there are few studies reporting different results concerning the cytotoxicity of silver. Inhibition of the proliferation of keratinocytes and fibroblasts after treatment with silver-sulfadiazin has been described [6, 7]. In contrast, Tweden et al. [3] have reported no evidence of silver toxicity to cultured fibroblasts until concentrations reached levels of 1200 ppb. In a previous animal trial we have reported about reduced infection rates of silver-coated diaphyseal implants in comparision of titanium implants in rabbits after artificial contamination with *S. aureus* without histologically proven cytotoxic local or systemic side-effects [9]. The stems of the implants have not yet been silver coated because of scepsis concerning the osseointegration. Though, a silver coating of prosthetic stems would be desirable in order to achieve antibactericidal silver concentrations in the bone.

Osseointegration is a process in which a stable anchorage of an implant in the bone is achieved by direct bone to implant contact without any intervening fibrous tissue [21, 22]. Osseointegration is influenced by various factors such as the used materials, design and size of the implant, and the surface characteristics of the medical device [21–24]. The basic prerequisite for osseointegration is the biocompatibility of the used material. Titanium has got well-known properties concern prostheses ingrowth in the bone, long term stability, tissue biocompactibility, and osseointegrative effects [22, 23]. Therefore titanium and titanium alloys are the most often used metals in modern cementless endoprosthetics often combined with a hydroxyapatite coating [22, 24, 25].

In literature there have been no studies describing osseointegration of silver-coated materials. Only a few reports describe the effect of silver on osteoblasts [10, 26]. Bosetti et al. [10] reported no cytotoxicity of silver-coated stainless steel on osteoblast-like cells in vitro determinded by cell morphology. They further described no decreased AP activity and no elevation of lactat dehydrogenase indicating a cell damage. Nevertheless, in this study no different silver concentrations have been used. Alt et al. [10] exposed human osteoblast cells (hFOB cell line) to bone cement with addition of nanoparticulate silver. They examined by fluorescence microscopy that all cells were vital and showed a typical mesh-like growth pattern after 48 hour incubation period. Kramer et al. [27] investigated the osteoinductive properties of silver on demineralized bone matrix implanted in the paraspinous muscles of rats. In this experiment, they described osteoinductive capacity of silver at low concentrations (10^−3^ M and 10^−4^ M) but not at higher concentrations (10^−5^ M). They concluded that there must be a concentration of silver which can instill a maximum antibacterial effect while retaining almost 100% of the osteoinductive capacity.

The current study confirms the results of Kramer et al. [27] concerning the dose-dependent effects of silver on osteoblasts in vitro. A silver supplement below 10 mg exhibits an increase of differentiation markers (AP activity) of osteoblastic phenotype indicating a stimulation of osteoblastic differentiation superior to a Ti-6Al-4V supplement. For the HOS-58 cell line the differences have been statistically significant (*P* < 0.5). At higher doses (>10 mg) silver caused lower AP activity in comparison to a Ti-6Al-4V supplement indicating lesser biocompatibility. Nevertheless, statistically differences could be observed at doses of 25 mg for the SAOS cell line only.

The reduction of the proliferation rate has been more pronounced with a silver supplement than with a Ti-6Al-4V supplement. At doses up to 10 mg of silver the reduced proliferation rate (40.4% for HOS-58) should not be seen as a cytotoxic effect, moreover it is believed to be a step in osteogenic maturation. This assumption is confirmed by increased AP activity at these doses indicating osteogenic differentiation. Nevertheless, at doses above 10 mg of silver the proliferation rate shows a more pronounced reduction combined with decreased markers of osteogenic differentiation. Therefore, at these doses silver should be seen as cytotoxic, particularly because of a proliferation rate below 10% for both cell lines. In contrast, titanium is known to have a low cytotoxicity [22]. Yao et al. [28] reported that the addition of titanium particles in low doses (4.5 × 10^7^ particles per milliliter) caused no reduced proliferation rate of osteosarcoma cell lines. In the current study, even with a 25 mg Ti-6Al-4V supplement the proliferation rate is still above 50% for both cell lines. Statistically significant differences beween the silver and the titanium group can still be detected in the SAOS cells at 10–25 mg supplements, whereas in the HOS-58 cells statistically significant differences can be observed at 20–25 mg only. The cytotoxic effect of silver seems to be more pronounced in high proliferative cell lines, whereas in cells with a lower proliferation rate (e.g., normal osteoblasts) this effect is less distinctive. Therefore, it can be assumed, that in normal osteoblasts the reduction of the proliferation rate at low silver doses will not inhibit osseointegration.

Osteocalcin as a marker for matrix mineralization did not show any differences between the two groups in the measured time period. The osteocalcine was expressed at low levels for all supplement concentrations in both cell lines. As a marker for the late phase of osteoblastic differentiation osteocalcin is found in mature and fully developed mineralized bone matrix. Our results seem to be an indicator of beginning bone turnover. Though osteocalcine production in osteosarcoma cell lines and especially in SAOS cells is controversial. Ahmad et al. [29] reported low amount of osteocalcine mRNA in SAOS-2 cultures treated with 1,25-(OH)_2_D_3_ for 10 weeks. Other studies reported simular results of low osteocalcine production in different osteosarcoma cells in in vitro studies [30].

Our results confirm the fact that silver at low concentrations is not cytotoxic for osteoblast in vitro. On the contrary, it seems to stimulate osteogenic maturation of undifferentiated osteosarcoma cells. The difficulty in using silver for antibacterial coating of prosthetic stems is the determination of the “therapeutic window” [26] in which silver acts bactericidal and additionally stimulates osteogenic differentiation. Nevertheless, statements concerning the osseointegrative properties of silver-coated stems can not be made yet, because osseointegration depends on many factors as stated above. We perform now an in vivo study, in which silver-coated versus titanium hip stems will be implanted in beagles. The osseointegration will be measured by RSA technique and importantly histologically after a follow-up period of twelve months.
